# Disease burden and attributable risk factors of major depressive disorder in China, Japan, and South Korea from 1990 to 2021 and its prediction to 2035

**DOI:** 10.3389/fpubh.2025.1510091

**Published:** 2025-04-14

**Authors:** Yifan Wang, Jingwen Zhu, Jihong Zhou

**Affiliations:** ^1^The Seventh Clinical College of Guangzhou University of Chinese Medicine, Shenzhen, China; ^2^The Fourth Clinical College of Guangzhou University of Chinese Medicine, Shenzhen, China

**Keywords:** China, Japan, and South Korea, GBD database, major depressive disorder, disease burden, age period cohort analysis, risk factors, Bayesian age-period-cohort

## Abstract

**Objective:**

To analyze the disease burden of major depressive disorder (MDD) and risk factors associated with MDD in China, Japan, and South Korea(CJK) from 1990 to 2021, to explore the effects of age, period, and cohort on the disease burden of MDD, and to predict the burden of MDD in CJK from 2021 to 2035.

**Methods:**

The Global Burden of Disease 2021 (GBD) database was searched to collect the incidence, prevalence, disability-adjusted life years (DALYs), and risk factors of MDD in CJK. The respondents were selected from the Global, China, Japan, and South Korea. The respondents’ disease was major depressive disorder. The respondents’ gender was male, female, and both. The time was selected from 1990 to 2021. The age was selected from the whole age group (less than 5 years old to over 95 years old). Trends in MDD burden from 1990 to 2021 were analyzed using Joinpoint 4.9.1.0. Age-period-cohort (APC) analyses were performed using the APC Web Tool. Bayesian age-period-cohort analyses (BAPC) were performed using R4.4.1.

**Results:**

Japan had the highest burden of MDD, followed by South Korea, and China had the lowest burden of MDD; The average annual percentage change (AAPC) of MDD burden indicators in China was less than 0, while in Japan and South Korea it was greater than 0. In 2021, middle-aged people aged 55–59 years had the highest burden of MDD in China, while in Japan and South Korea, young people aged 20–29 years had the highest burden of MDD, and the burden of MDD was higher among females than males in CJK from 1990 to 2021; APC analyses showed that the net drifts, local drifts, and RR values of the MDD burden indicators in CJK in terms of age, period, and cohort effects were closely related to the economic forms and aging populations; MDD had 3 levels and 5 risk factors; The burden of MDD in China would be in a decreasing trend between 2021 and 2035, and the burden of MDD in Japan and South Korea would be in a decreasing trend between 2021 and 2022, and an increasing trend between 2022 and 2035.

**Conclusion:**

There are both commonalities and differences in the burden of MDD in CJK, while the current burden of MDD in CJK is lower than the global average. Compared with Japan and South Korea China has a lower burden of MDD, but all three countries still face enormous challenges in the prevention and control of MDD.

## 1 Introduction

Major depressive disorder (MDD) is a common chronic disease with persistent and significant depressed mood as the main clinical feature (1). About 300 million people are affected by MDD globally, which is predicted to become the first global disease burden by 2030 according to the World Health Organization (WHO) (2). Not only that, the WHO report shows that depression and anxiety disorder cause about 12 billion working days loss worldwide every year, with an annual loss of up to 1 trillion US dollars (3). The study found that up to 60% of suicides suffer from MDD, and the suicide mortality of patients with depression is about 20 times that of the general population (4). In addition, depression is closely related to a series of health problems such as cardiovascular disease, cancer, diabetes, and respiratory diseases (5, 6). Affected by COVID-19 and the global economic recession after the epidemic, people’s pressure on their health, life, and studies is still increasing. Therefore, how to effectively prevent and control MDD is a worldwide public health issue (7).

Due to the influence of geographic factors, economic development, and historical and cultural traditions, CJK is facing some similar problems and dilemmas in the process of modernization. In terms of culture, CJK are all deeply influenced by Confucian culture. On the one hand, Confucian thoughts of benevolence, righteousness, propriety, wisdom, and faithfulness have profoundly shaped the cultural and social structure of these countries. On the other hand, Confucian ideological pressures such as “restrain yourself and follow social norms,” “three cardinal guides and five constant virtues” and “men are superior to women” have to some extent stifled the expression of individual personality and exacerbated class solidification and gender inequality (8). In terms of social economy, the three countries have experienced rapid economic growth and decline after World War II, with relatively similar economic development models, and they all rely on export-oriented economies. The intensification of global competition has led to serious “Involution” in the three countries (9). In terms of academics, all three countries attach great importance to teenagers’ education and have very strict examination systems. Teenagers in the three countries are faced with a huge academic burden (10).

The GBD database covers indicators of MDD incidence, prevalence, mortality, DALYs, etc. (11). Therefore, this study quantifies the overall burden of MDD in CJK based on the GBD database and predicts the future burden of MDD in the three countries to provide focus and direction for the prevention and treatment of MDD in the relevant countries, as well as data support for health policy decision makers to accurately and efficiently allocate healthcare resources, identify high-risk populations, and formulate prevention strategies.

## 2 Materials and methods

### 2.1 Data sources

The data in this study were based on the GBD 2021 database published by the Institute for Health Metrics and Evaluation at the University of Washington, USA, which provides a comprehensive assessment of 371 diseases and 88 risk factors in 204 countries and territories (12). GBD 2021 database includes over 80,000 different data sources to produce the most scientifically rigorous estimates possible (13).

### 2.2 Research methods

This study classified the diseases studied using the International Classification of Diseases Tenth Edition (ICD-10) and the Diagnostic and Statistical Manual of Mental Disorders Fifth Edition (DSM-V). The respondents were selected from the Global, China, Japan, and South Korea. The respondents’ disease was major depressive disorder. The respondents’ gender was male, female, and both. The time was selected from 1990 to 2021. The age was selected from the whole age group (less than 5 years old to over 95 years old). The GBD Outcomes Using the ASIR, ASPR, and ASDR of MDD as indicators for measuring MDD burden, and to calculate the AAPC for ASIR, ASPR, and ASDR for CJK from 1990 to 2021. The data of incidence rate, prevalence, and DALYs of depression in CJK from 1990 to 2021 were summarized by sex and age group. The age-period-cohort (APC) model was used to estimate the net drifts, local drifts of the age model, and the rate ratio (RR) of the period and cohort models (14). Risk factors for MDD were extracted from the GBD database, and population-attributable risk (PAF) was used to indicate the degree of harm caused by exposure to risk factors to the entire population. BAPC was used to predict ASIR, ASPR, and ASDR of MDD from 2021 to 2035.

### 2.3 Statistical method

Data were organized using Excel 2023 and the tidyverse and reshape2 packages in R 4.4.1. AAPC analysis was performed using Joinpoint 4.9.1.0. APC Web Tool^1^ was used for APC analysis. BAPC analysis was performed using BAPC with the INLA package. Data were visualized and analyzed using the GBDR package, ggpubr package, and ggplot2 package.

## 3 Results

### 3.1 Disease burden situation

**Figure 1:**
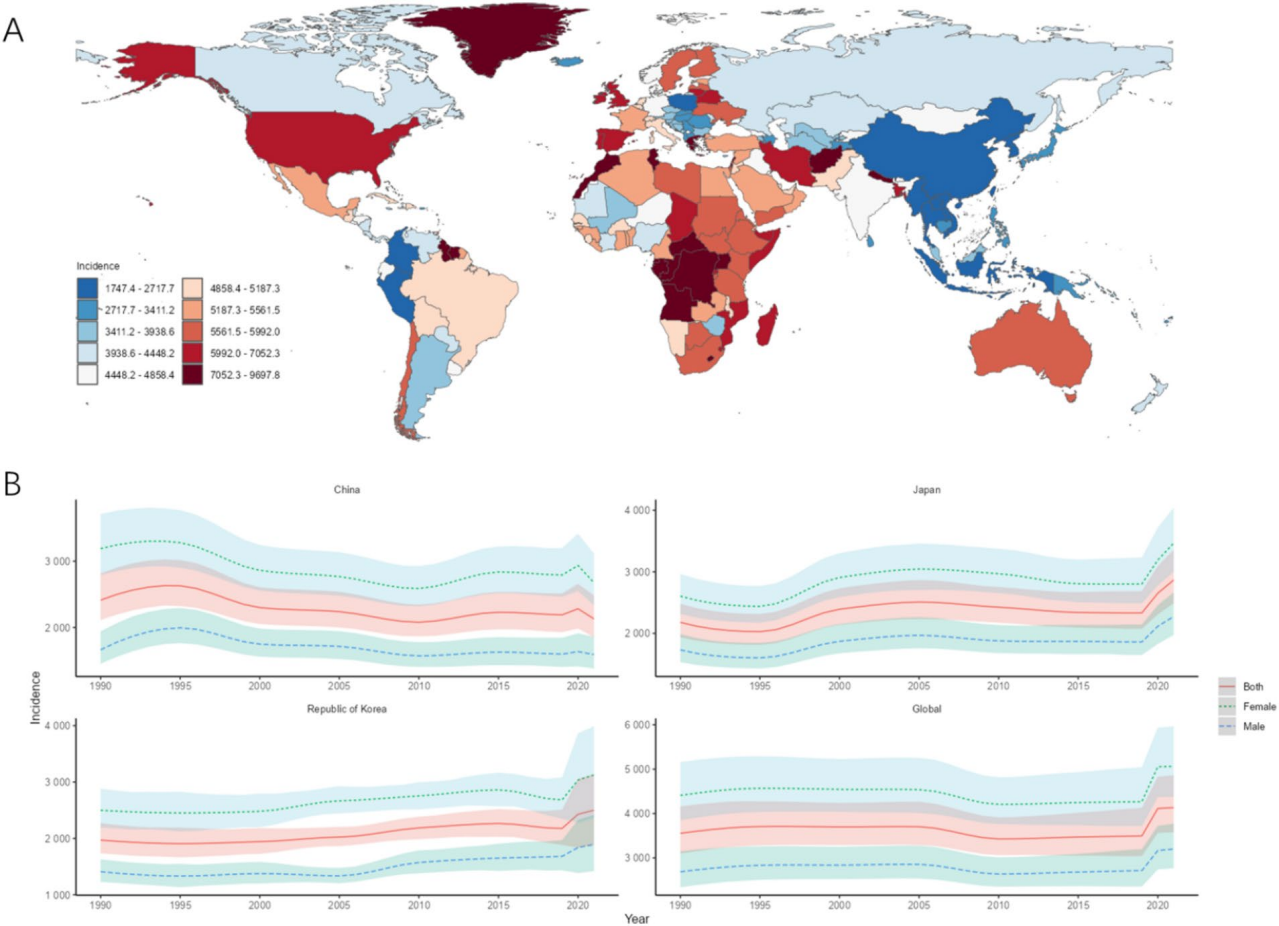
ASIR of MDD. **(A)** ASIR for MDD in 2021 for 204 countries and territories; **(B)** ASIR time trend for MDD. The blue line and its surrounding area represent the prediction curves and uncertainty intervals for males; the green line and its surrounding area represent the prediction curves and uncertainty intervals for females; and the red line and its surrounding area represent the prediction curves and uncertainty intervals for both sexes in total.

The highest ASIR in CJK in 2021 was Japan [2867.42/100,000 (95% UI: 2491.24, 3358.04)], followed by South Korea [2500.36/100,000 (95% UI: 1881.61, 3139.53)], and the lowest was in China [2129.47/100,000 (95% UI: 1854.28, 2481.48)]. The ASIR of MDD in CJK was lower than the world average of 4135.11/100,000 (95% UI, 3580.46, 4865.00), and the ASIR of MDD in CJK and globally in 1990–2021 was higher in females than in males. Compared with 1990, the AAPC of ASIR in China is less than 0, while the AAPCs of ASIR in Japan, South Korea, and globally are all greater than 0, as shown in Figures 1A,B and Table 1.

**Table 1:** ASIR and trends of MDD in CJK.

Country	Age-standardized incidence per 100,000 population (95%UI)		AAPC (95%CI)
	1990	2021	
China	2412.43 (2112.78, 2813.35)	2129.47 (1854.28, 2481.48)	−6.43 (−8.65, −4.22)
Japan	2179.04 (1935.09, 2479.11)	2867.42 (2491.24, 3358.04)	23.29 (21.54, 25.04)
South Korea	1970.43 (1736.55, 2269.91)	2500.36 (1881.61, 3139.53)	17.50 (13.63, 21.36)
Global	3554.71 (3100.09, 4160.30)	4135.11 (3580.46, 4865.00)	19.55 (15.89, 23.20)

**Figure 2:**
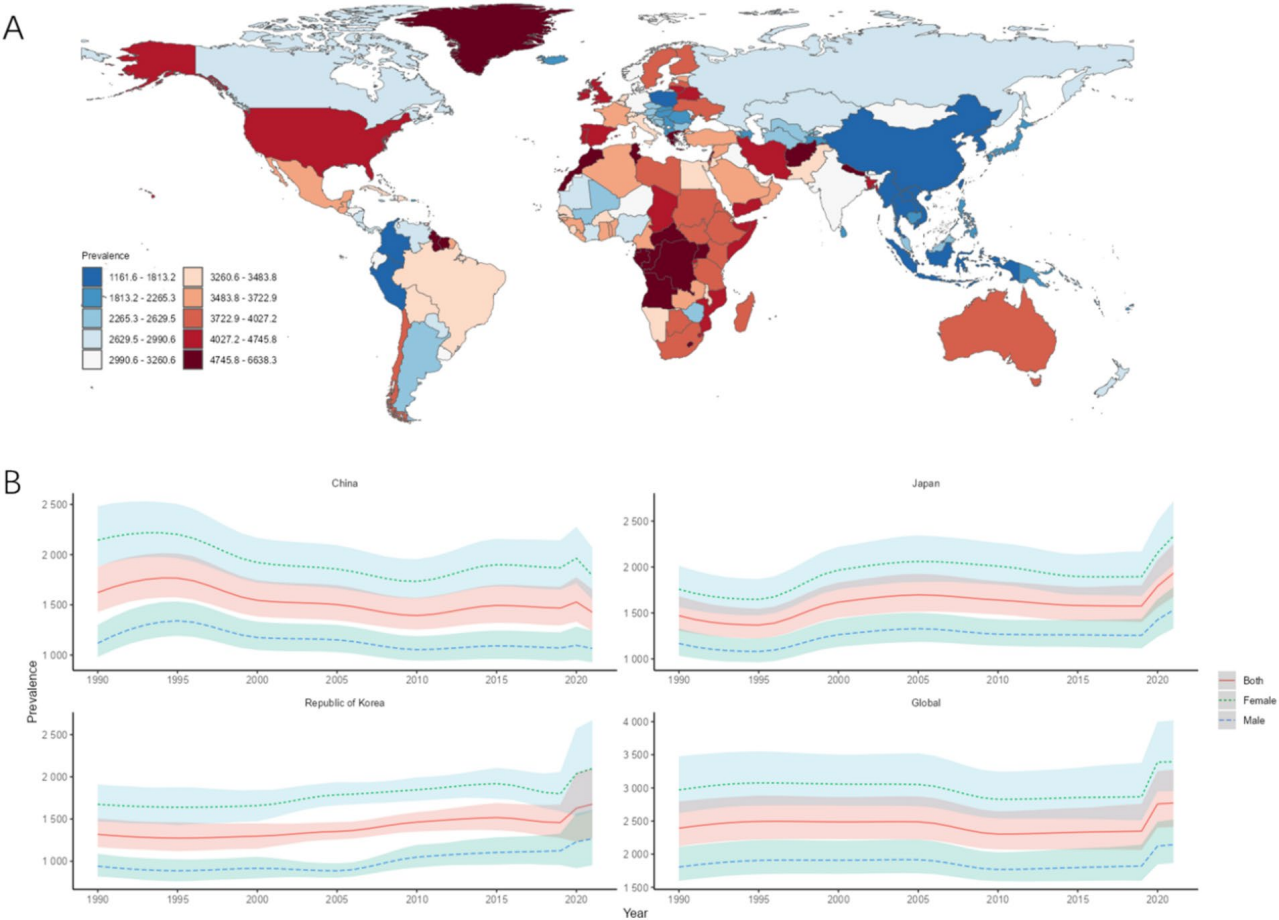
ASPR of MDD. **(A)** ASIR for MDD in 2021 for 204 countries and territories; **(B)** ASPR time trend for MDD. The blue line and its surrounding area represent the prediction curves and uncertainty intervals for males; the green line and its surrounding area represent the prediction curves and uncertainty intervals for females; and the red line and its surrounding area represent the prediction curves and uncertainty intervals for both sexes in total.

The country with the highest ASPR in CJK in 2021 was Japan [1933.07/100,000 (95% UI: 1675.60, 2250.65)], followed by South Korea [1675.15/100,000 (95% UI: 1270.11, 2103.35)], and the lowest was China [1426.49/100,000 (95% UI: 1241.64, 1653.11)]. The ASPR of MDD in CJK was lower than the world average of 2771.64/100,000 (95% UI: 2412.59, 3273.68), and the ASPR of MDD in CJK and globally in 1990–2021 was higher in females than in males. Compared with 1990, the AAPC of ASPR in China is less than 0, while the AAPCs of ASPR in Japan, South Korea, and globally are all greater than 0, as shown in Figures 2A,B and Table 2.

**Table 2:** ASPR and trends of MDD in CJK.

Country	Age-standardized prevalence per 100,000 population (95%UI)		AAPC (95%CI)
	1990	2021	
China	1622.13 (1427.65, 1880.26)	1426.49 (1241.64, 1653.11)	−4.28 (−5.91, −2.65)
Japan	1470.12 (1307.33, 1682.13)	1933.07 (1675.60, 2250.65)	15.67 (14.47, 16.86)
South Korea	1318.23 (1167.86, 1505.71)	1675.15 (1270.11, 2103.35)	12.75 (11.74, 13.75)
Global	2392.51 (2118.09, 2793.54)	2771.64(2412.59, 3273.68)	14.29 (10.45, 18.14)

**Figure 3:**
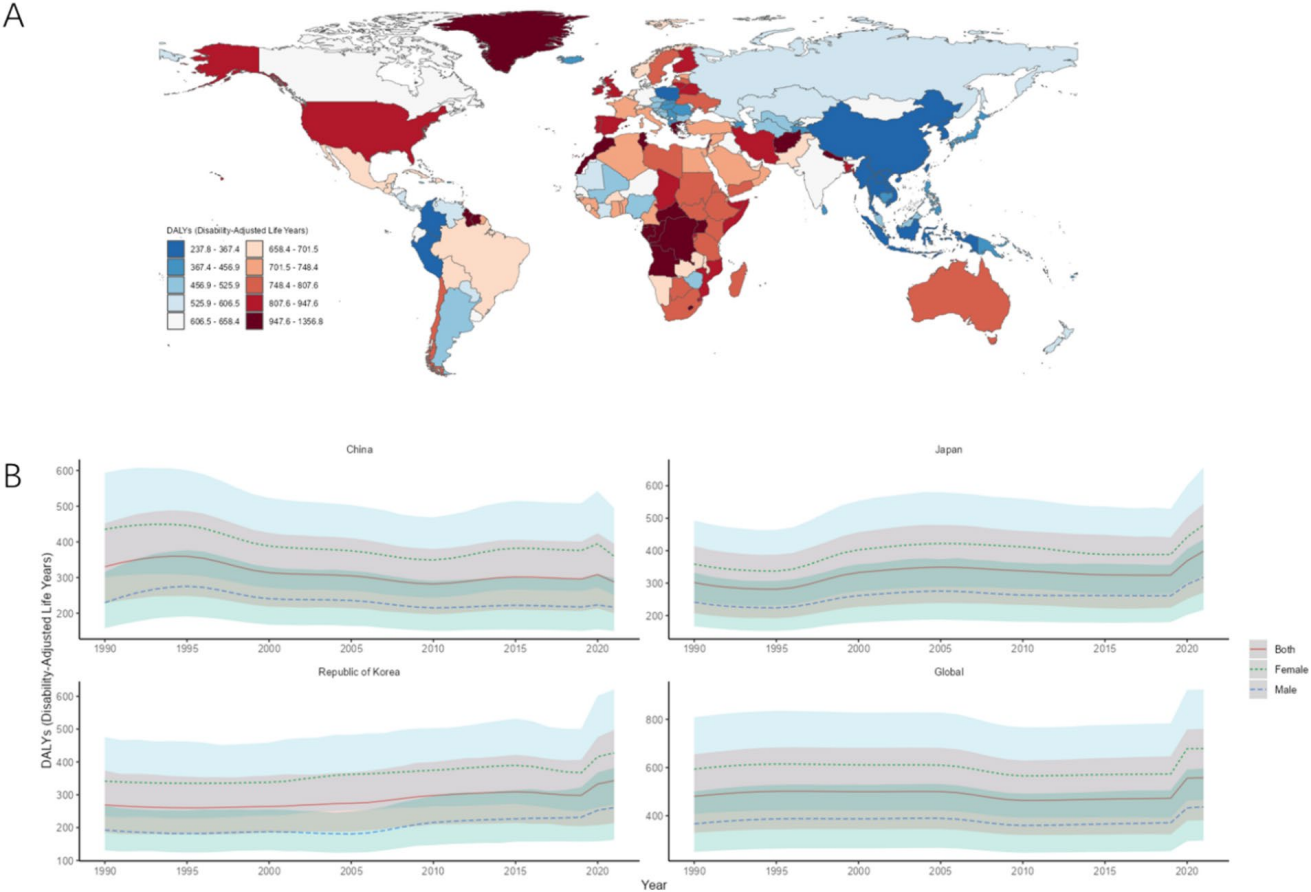
ASDR of MDD. **(A)** ASDR for MDD in 2021 for 204 countries and territories; **(B)** ASDR time trend for MDD. The blue line and its surrounding area represent the prediction curves and uncertainty intervals for males; the green line and its surrounding area represent the prediction curves and uncertainty intervals for females; and the red line and its surrounding area represent the prediction curves and uncertainty intervals for both sexes in total.

The highest ASDR in CJK in 2021 was Japan [398.40/100,000 (95% UI: 271.18, 544.40)], followed by South Korea [342.80/100,000 (213.93, 497.60)], and the lowest was China [287.48/100,000 (200.08, 394.39)]. The ASDR of MDD in CJK is below the world average of 557.87/100,000 (381.20, 760.70), and the ASDR of MDD in CJK and globally in 1990–2021 was higher in females than in males. Compared with 1990, the AAPC of ASDR in China is less than 0, while the AAPCs of ASDR in Japan, South Korea, and globally are all greater than 0, as shown in Figures 3A,B and Table 3.

**Table 3:** ASDR and trends of MDD in CJK.

Country	Age-standardized DALYs per 100,000 population (95%UI)		AAPC (95%CI)
	1990	2021	
China	330.26 (227.84, 451.47)	287.48 (200.08, 394.39)	−1.02 (−1.32, −0.71)
Japan	301.35 (207.02, 413.78)	398.40 (271.18, 544.40)	3.28 (3.03, 3.53)
South Korea	268.96 (185.01, 373.76)	342.80 (213.93, 497.60)	2.67 (2.45, 2.88)
Global	480.63 (329.43, 654.90)	557.87 (381.20, 760.70)	2.91 (2.14, 3.69)

### 3.2 Age and gender differences

**Figure 4:**
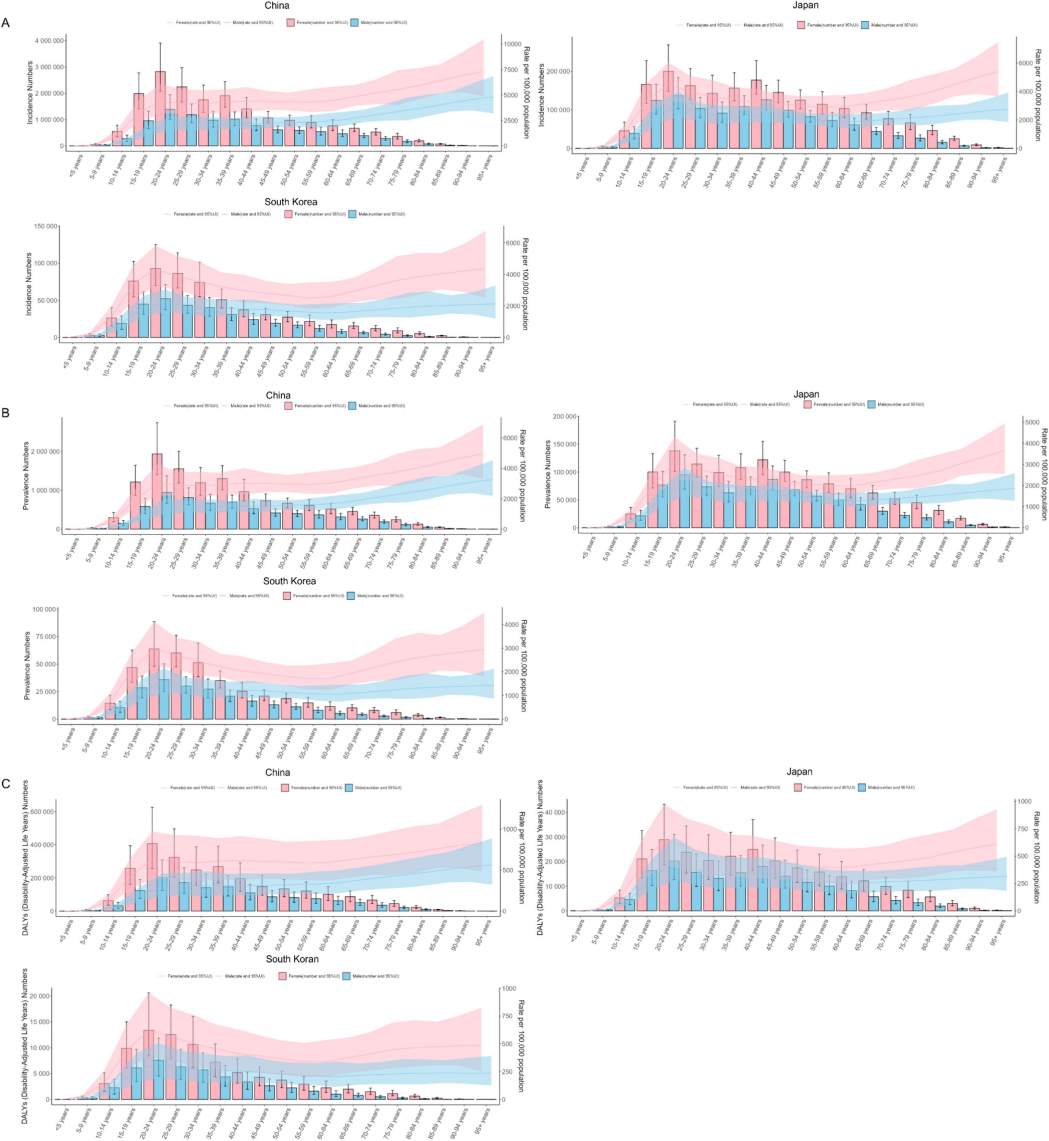
Burden of MDD in CJK in 1990. **(A)** Incidence; **(B)** Prevalence; **(C)** DALYs. Pink bars and pink areas represent the burden of disease and uncertainty interval for females; blue bars and blue areas represent the burden of disease and uncertainty interval for males.

**Figure 5:**
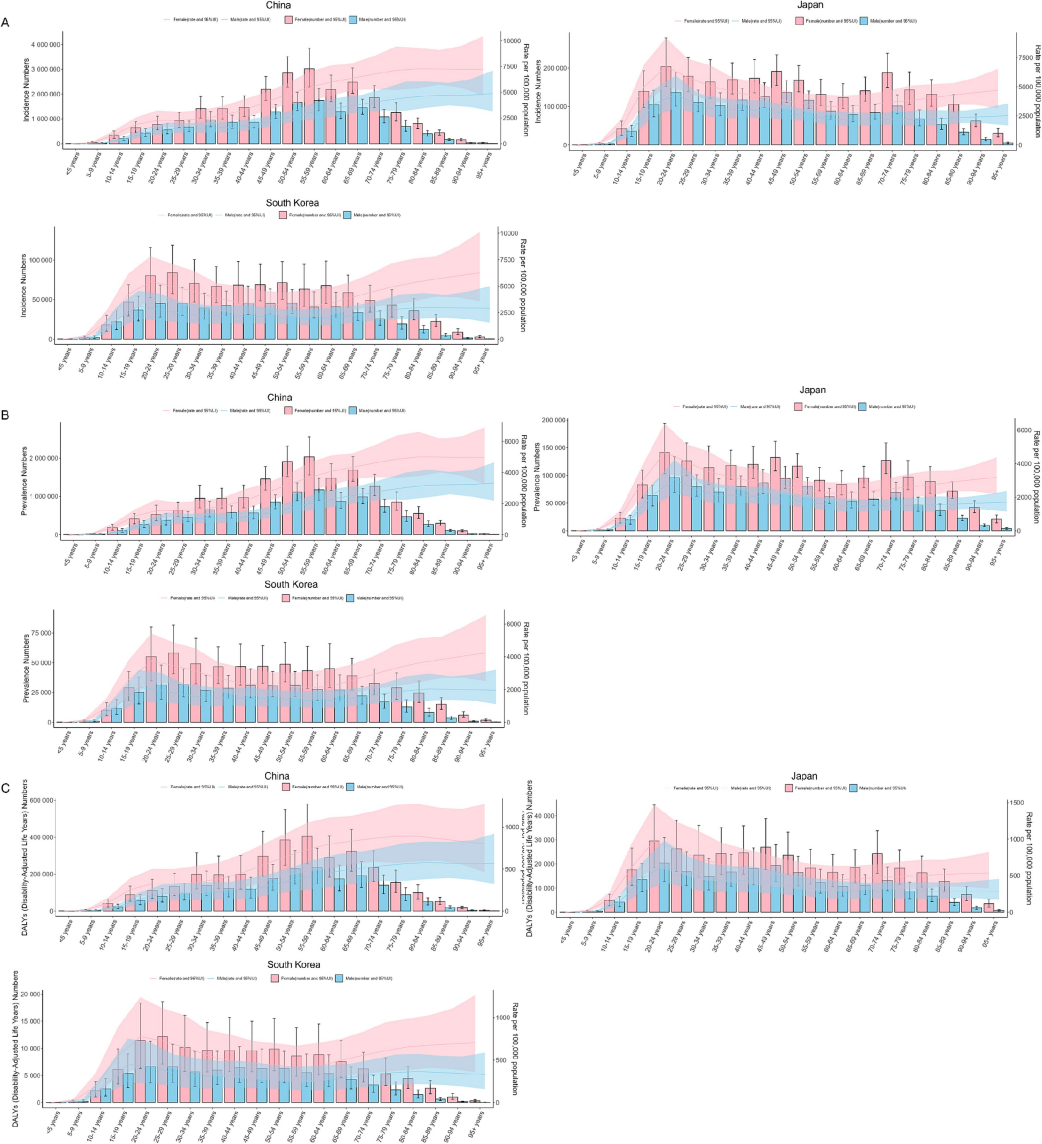
Burden of MDD in CJK in 2021. **(A)** Incidence; **(B)** Prevalence; **(C)** DALYs. Pink bars and pink areas represent the burden of disease and uncertainty interval for females; blue bars and blue areas represent the burden of disease and uncertainty interval for males.

In 1990, the incidence, prevalence, and DALYs were higher for females than for males in all age groups in CJK. The highest incidence rate, prevalence rate, and DALYs of males and females in CJK were young people aged 20–24, as shown in Figures 4A–C. In 2021, the incidence, prevalence, and DALYs of females in all age groups were also higher for females than for males in all age groups in CJK. The highest incidence, prevalence, and DALYs in 2021 for males and females in Japan and South Korea were in young adults aged 20–29, and in China, they were highest in middle-aged adults aged 55–59, as shown in Figures 5A–C.

### 3.3 Age-period-cohort model analysis

**Figure 6:**
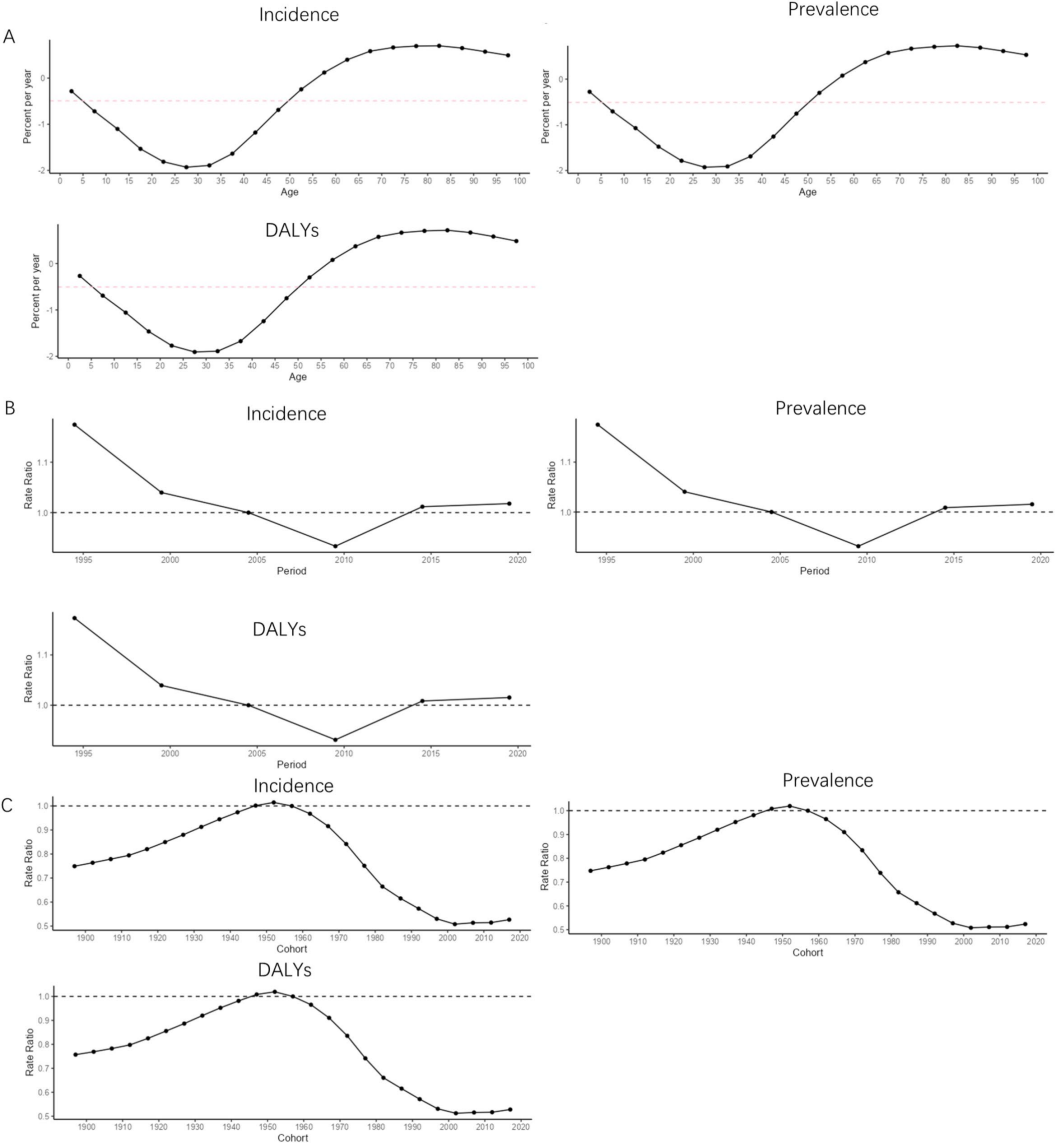
APC trend of MDD burden in China from 1990 to 2021. **(A)** Age effect; **(B)** Period effect; **(C)** Cohort effect.

The net drift of MDD incidence [−0.49 (−0.78−0.21)], prevalence [−0.51 (0.86−0.16)], and DALYs [−0.50 (0.86−0.15)] in China was less than 0 for the period 1990–2021 in terms of age effects. In the age range of 0–30 years, the local drifts of the MDD burden indicators in China showed a decreasing trend with age. In the age range of 30–80 years, the local drifts of the MDD burden indicators in China showed an increasing trend with age. After the age of 80 years, the local drifts of the MDD burden indicators in China showed a decreasing trend with age, as shown in Figure 6A. From 1990 to 2021, in terms of period effect, the RR values of MDD burden indicators in China all showed a continuous decreasing trend during 1990–2009, and showed a continuously increasing trend after 2010, as shown in Figure 6B. During the period 1990–2021 on the cohort effect, the RR values of China’s MDD burden indicators all showed an increasing trend before 1955, all showed a decreasing trend during 1955–1999, and all showed an increasing trend after 2000, as shown in Figure 6C.

**Figure 7:**
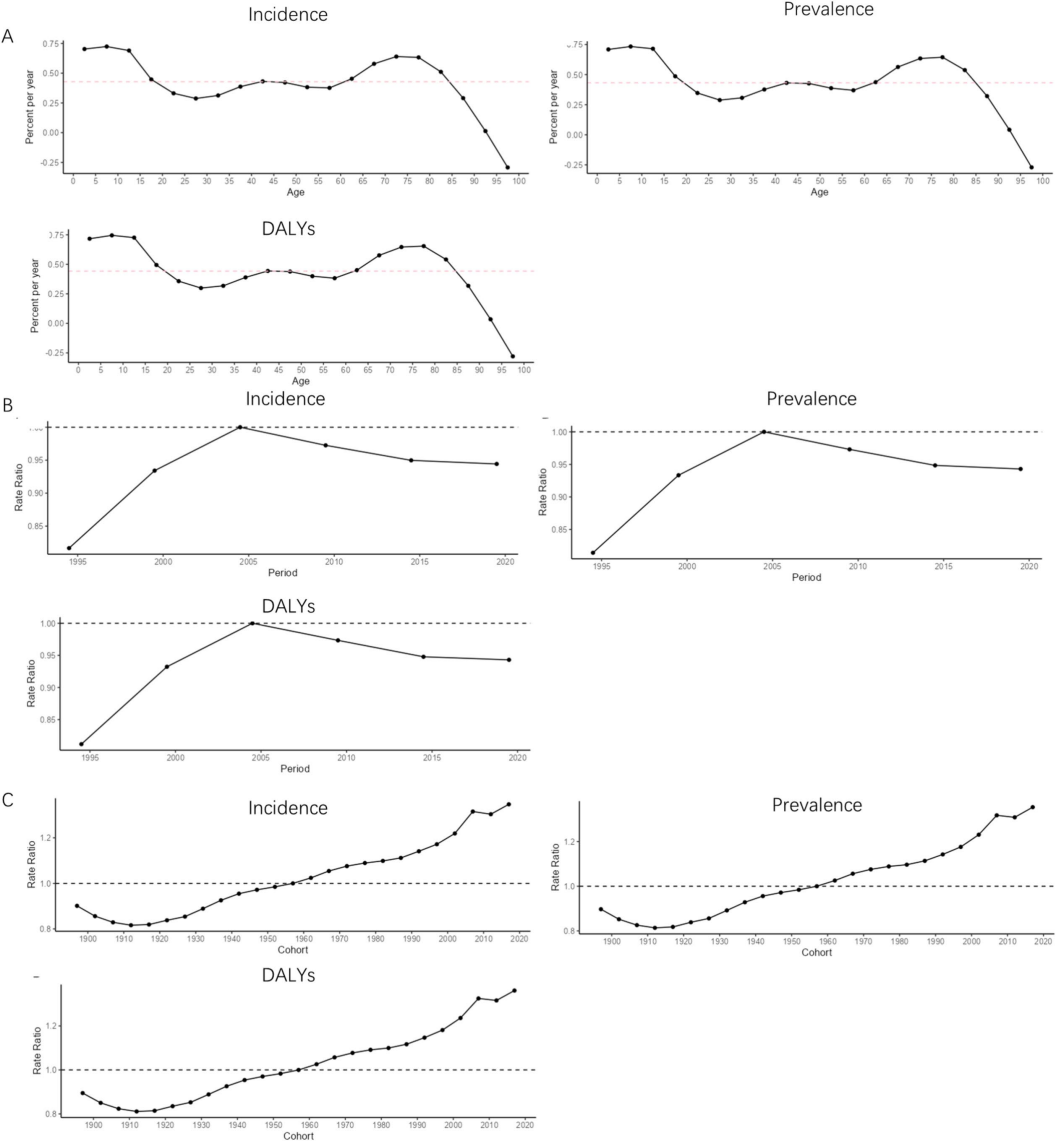
APC trend of MDD burden in Japan from 1990 to 2021. **(A)** Age effect; **(B)** Period effect; **(C)** Cohort effect.

The net drift of MDD incidence [0.43 (0.16, 0.69)], prevalence [0.43 (0.08, 0.78)], and DALYs [0.44 (0.11, 0.77)] in Japan was greater than 0 for the period 1990–2021 in terms of age effects. In the age range of 0–10 years, the local drifts of the MDD burden indicators in Japan showed an increasing trend with age. In the age range of 10–30 years, the local drifts of the MDD burden indicators in Japan showed a decreasing trend with age. In the age range of 30–45 years, the local drifts of the MDD burden indicators in Japan showed an increasing trend with age. In the age range of 45–60 years, the local drifts of the MDD burden indicators in Japan showed a decreasing trend with age. In the age range of 60–80 years, the local drifts of MDD prevalence and DALYs in Japan tended to increase with increasing age, and in the age range of 60–75 years, the local drifts of MDD incidence tended to increase with age. After the age of 80, the local drifts of MDD prevalence and DALYs in Japan showed a decreasing trend with age. After the age of 75, the local drifts of MDD incidence in Japan showed a decreasing trend with age, as shown in Figure 7A. For the period 1990–2021 in terms of period effects, the RR values of the MDD burden indicator in Japan showed a continuous upward trend in 1990–2004 and a continuous downward trend in the post-2005 period, as shown in Figure 7B. For the period 1990–2021 in terms of cohort effects, the RR values of Japan’s burden of disease indicators showed a continuous downward trend until 1914, an upward trend during 1915–2004, a downward trend after 2005–2014, and an upward trend after 2015, as shown in Figure 7C.

**Figure 8:**
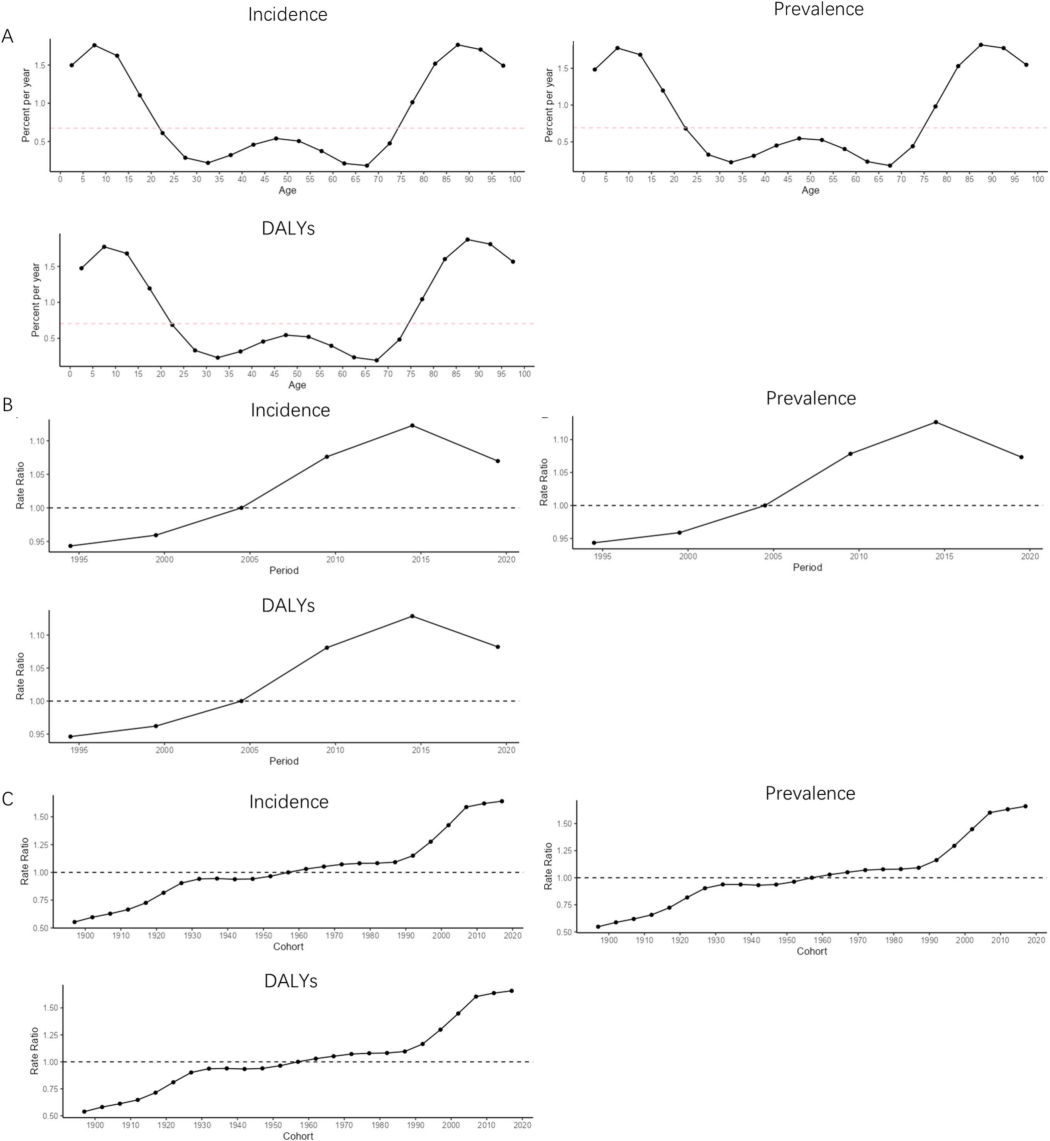
APC trend of MDD burden in South Korea from 1990 to 2021. **(A)** Age effect; **(B)** Period effect; **(C)** Cohort effect.

The net drift of MDD incidence [0.67 (0.20, 1.15], prevalence [0.69 (0.11, 1.27], and DALYs [0.70 (0.14, 1.27] in South Korea was greater than 0 for the period 1990–2021 in terms of age effects. In the age range of 0–10 years, the local drifts of the MDD burden indicators in South Korea showed an increasing trend with age. In the age range of 10–35 years, the local drifts of the MDD burden indicators in South Korea showed a decreasing trend with age. In the age range of 35–50 years, the local drifts of the MDD burden indicators in South Korea showed an increasing trend with age. In the age range of 50–70 years, the local drifts of the MDD burden indicators in South Korea showed a decreasing trend with age. In the age range of 70–90 years, the local drifts of the MDD burden indicators in South Korea showed an increasing trend with age. After the age of 90 years, the local drifts of the MDD burden indicators in South Korea showed a decreasing trend with age, as shown in Figure 8A. For the period 1990–2021 in terms of period effects, the RR values of the MDD burden indicator in South Korea showed a continuous upward trend in 1990–2014 and a continuous downward trend in the post-2015 period, as shown in Figure 8B. For the period 1990–2021 in terms of cohort effects, the RR values of South Korea’s burden of MDD indicators showed a continuous upward trend until 1934, a downward trend during 1935–1939, and an upward trend after 1940, as shown in Figure 8C.

### 3.4 Risk factors

**Figure 9:**
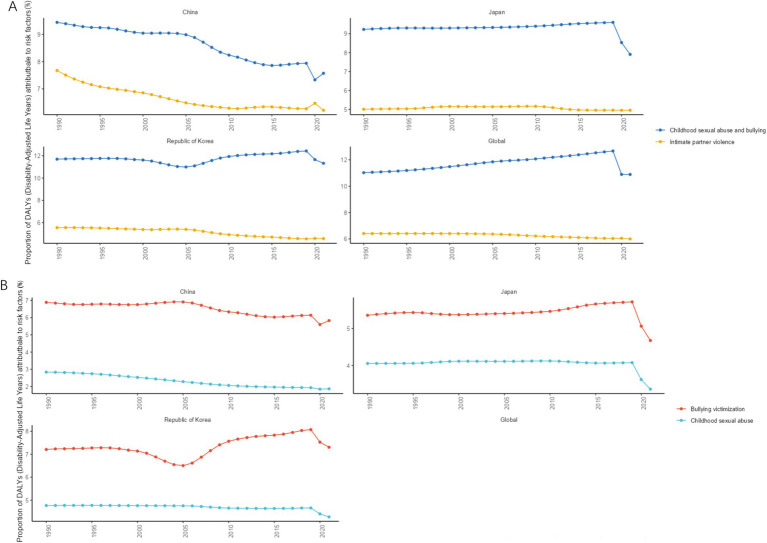
Changes in PAF of MDD from 1990 to 2021. **(A)** Level 2 risk factors; **(B)** Level 3 risk factors.

The GBD 2021 database systematically assessed the impact of 88 risk factors on health outcomes globally and classified disease risk factors at four levels. The first level covered three major categories of risk factors: environmental/occupational risks, behavioral risks, and metabolic risks. The subsequent risk factors were further subdivided based on first-level risk factors (15). The different levels of MDD risk factors obtained in this study were as follows: level 1 risk factors included behavioral risks; level 2 risk factors included childhood sexual abuse and bullying as well as intimate partner violence; and level 3 risk factors included childhood sexual abuse as well as bullying victimization. From 1990 to 2021, the top risk factors among the level 2 risk factors in CJK were child sexual abuse and bullying, followed by intimate partner violence, as shown in Figure 9A and Table 4. The top risk factor among the level 3 risk factors was bullying victimization, followed by child sexual abuse, as shown in Figure 9B and Table 4.

**Table 4:** Main risk factors for MDD in CJK in 1990 and 2021.

Level	Risk factor	Country	Age-standardized rate per 100000 (95%UI)	
			1990	2021
1	Behavioral risk	China	55.09 (94.89, 21.64)	38.86 (69.82, 15.54)
1	Behavioral risk	Japan	41.81 (73, 18.1)	50.1 (87.25, 21.18)
1	Behavioral risk	South Korea	45.15 (75.72, 20.74)	52.69 (90.15, 24.97)
2	Childhood sexual abuse and bullying	China	31.3 (55.68, 15.38)	21.83 (39.39, 10.33)
2	Childhood sexual abuse and bullying	Japan	27.9 (48.63, 14.25)	31.58 (54.98,15.79)
2	Childhood sexual abuse and bullying	South Korea	31.53 (54.4, 15.47)	38.47 (66.68, 19.57)
2	Intimate partner violence	China	25.43 (57.12, 0.07)	17.91 (41.05, 0.02)
2	Intimate partner violence	Japan	15.11 (34.99,0.02)	19.84 (45.46, 0.02)
2	Intimate partner violence	South Korea	15.01 (36.46, 0.05)	15.65 (37.65, 0.06)
3	Bullying victimization	China	22.83 (46.51, 8.92)	16.81 (33.01,6.94)
3	Bullying victimization	Japan	16.21 (33.5, 6.12)	18.69 (38.46, 7.27)
3	Bullying victimization	South Korea	19.46 (40.61, 7.51)	24.82 (50.13, 9.37)
3	Childhood sexual abuse	China	9.4 (16.75, 4.49)	5.37 (9.51, 2.55)
3	childhood sexual abuse	Japan	12.23 (20.03, 5.8)	13.4 (22.85, 6.18)
3	Childhood sexual abuse	South Korea	12.83 (21.05, 6.1)	14.52 (24.59, 7.06)

### 3.5 Prediction of disease burden trends

**Figure 10:**
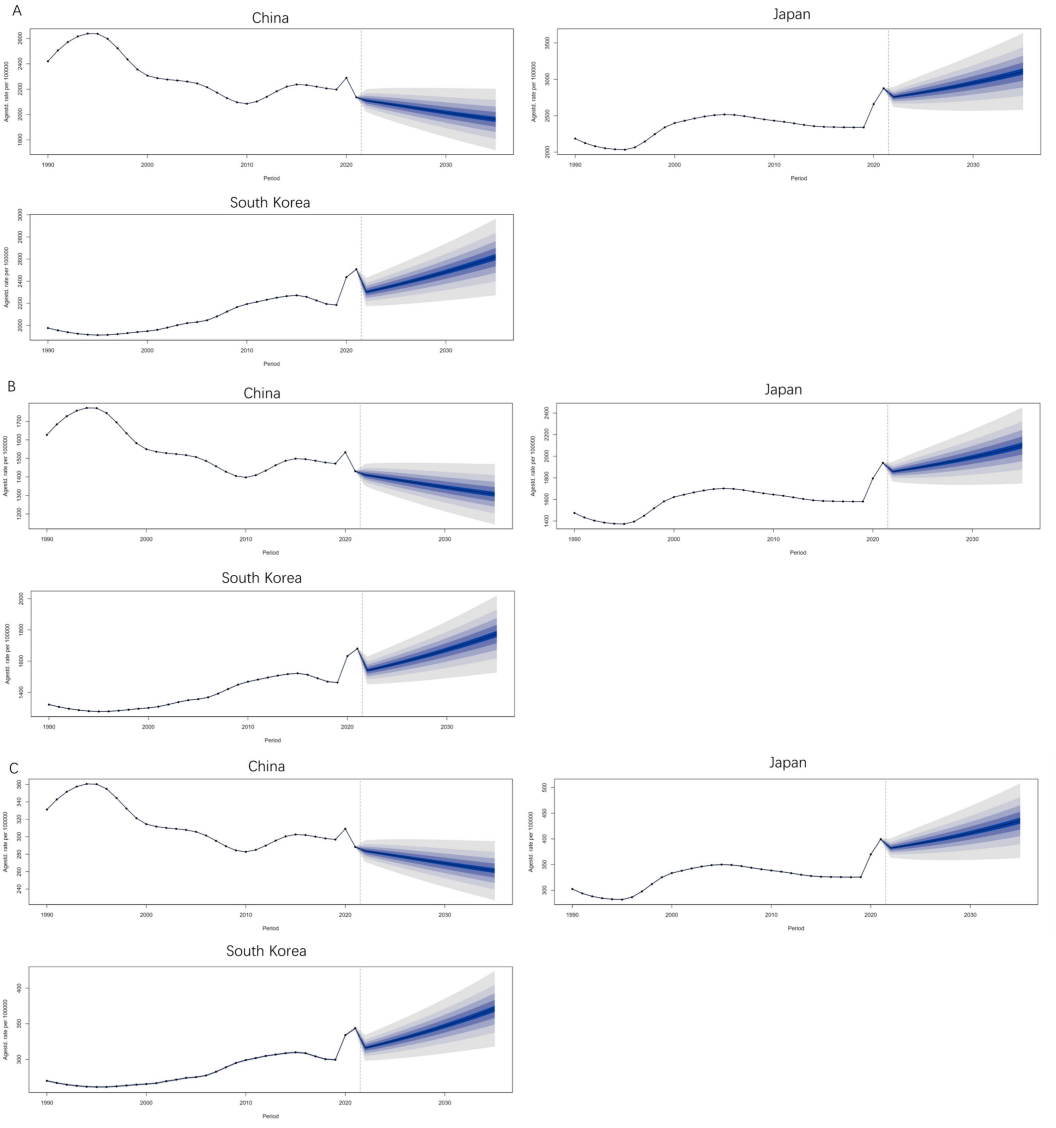
Prediction of Burden Trend of MDD from 2021 to 2035. **(A)** ASIR; **(B)** ASPR; **(C)** ASDR.

BAPCs predict that by 2035, the ASIR [3106.85/100000 (95% UI: 2483.78, 3299.92)], ASPR [2099.09/100,000 (95% UI: 1681.53, 2516.65)], and ASDR [598.99/100,000 (95% UI: 488.81, 709.16)] of MDD in Japan will be the highest among the three countries. South Korea’s ASIR [2617.98/100,000 (95%UI: 2208.063027.90)], ASPR [1773.90/100,000 (95%UI:1481.942065.85)], and ASDR [370.83/100,000 (95%UI:308.08433.57)] will be in second place. China’s ASIR [1962.23/100,000 (95%UI:1675.93 2248.54)], ASPR [1306.72/100,000 (95%UI:1113.76 1499.68)], and ASDR [261.16 /100,000 (95%UI: 221.09 301.24)] will be the lowest among CJK. The ASIR, ASPR, and ASDR of MDD in China will be in a decreasing trend during 2021–2035, and the ASIR, ASPR, and ASDR of MDD in Japan and South Korea will be in a decreasing trend during 2021–2022 and will be in an increasing trend during 2022–2035, as shown in Figures 10A–C.

## 4 Discussion

CJK is geographically close to each other, and all three countries are major countries in East Asia (16). Culturally CJK all belong to the same Chinese character culture circle, and they have similar cultural backgrounds (17). Confucian culture has a profound influence on CJK. Influenced by the values of restraint and harmony in Confucian culture, China, Japan, and South Korea all have some suppression of personal emotions and psychological states (18). Economically, all three countries are either economically developed or rapidly developing, facing similar issues such as fast work pace, intense competition pressure, and accelerated population aging trends.

China’s ASIR, ASPR, and ASDR of MDD were the lowest among the three countries, and the AAPCs of China’s ASIR, ASPR, and ASDR of MDD were less than 0 from 1990 to 2021. The BAPCs projections showed that the ASIR, ASPR, and ASDR of MDD in China would be in a decreasing trend from 2021 to 2035. The present study showed that the burden indicator of MDD in China’s young population aged 15–39 years declined particularly significantly between 1990 and 2021, which may be attributed to the rapid economic growth in China during the period of 1990–2019, which led to the improvement of people’s living standards, thus improving the mental health of young people to some extent (19, 20). Meanwhile, in recent years, the implementation of a series of policies and regulations targeting the mental health of adolescents has promoted the popularization of mental health education services, which may help to enhance the mental health status of young people (21). According to the present study, the burden of MDD was heaviest among middle-aged people aged 55–59 years in China, and according to the trend analysis of the APC age effect coefficient, the burden of disease of MDD in the age group of 55 years and above in China was on an increasing trend (local drift>0) from 1990 to 2021. This may be due to the rapid development of the Chinese economy, which has led to intensified job competition, increased working hours, and unequal income distribution among middle-aged people in China. At the same time, middle-aged people in China were also facing the emergence of chronic diseases, career development bottlenecks, children leaving home, and issues such as caring for their parents and their deaths (22). Meanwhile, the burden of MDD on China’s older adult population was also increasing due to factors such as population aging, an increase in the number of migrant workers in urban areas, and an increase in the number of empty nesters (23–25). According to the trend of APC time effect coefficients, the burden of MDD in China was in a rapidly decreasing trend between 1995 and 2009, while the disease burden of MDD was in an increasing trend after 2010. The average annual growth rate of China’s GDP during the 15 years from 1995 to 2009 was close to 10% (26). China’s economic takeoff has greatly improved people’s living standards and living conditions, thus helping to reduce the burden of MDD, but after 2010, China’s economic growth rate slowed down and was also affected by the subprime mortgage crisis and the COVID-19 outbreak, the burden of MDD disease in China began to increase after 2010 (27).

The ASIR, ASPR, and ASDR of MDD in Japan were the highest among the three countries, and the AAPCs of the ASIR, ASPR, and ASDR of MDD in Japan for the period 1990–2021 were greater than 0. The BAPCs projections showed that the ASIR, ASPR, and ASDR of MDD in Japan would be in an upward trend for the period 2021–2035. In the early 1990s, Japan’s bubble economy collapsed, and economic development entered a prolonged downturn, with the 1990s widely regarded as “Japan’s lost decade.” From 2000 to 2007, the Japanese economy grew positively for eight consecutive years. However, under the impact of the international financial crisis, the Japanese economy showed negative growth again in 2008–2009. From 2010 to 2018, after the Japanese economy resumed positive growth, in 2019, the Japanese economy experienced negative growth again due to the impact of COVID-19 (28). Over the past 30 years, the Japanese job market has become rigid, the wealth gap has widened, social classes have become more rigid, and the problems of aging and low birth rates have continued to intensify. At the individual level, life difficulties, psychological pressure, overwork culture, and mental health issues have become increasingly prominent (29). These factors may have led to the increase in the burden of MDD in Japan from 1990 to 2021. Although the Japanese economy was in the doldrums during these 30 years, Japan remained at the level of a developed country in many respects (30), and thus the burden of MDD in Japan remained below the world average. For example, in terms of medical security for the older adult, Japan promulgated the older adult welfare act in 1970. With the development of aging, the number of bedridden and disabled older adult people has increased accordingly. In 1982, Japan passed the older adult health care law, which not only stipulates the medical security of the older adult but also attaches importance to the health care of the older adult. In 2000, Japan established a care insurance system to provide care services for the older adult who need care. Japan’s perfect medical security system for the older adult has improved the quality of life of the older adult and reduced the spiritual burden of the older adult to a certain extent (31). According to this study, young people aged 20–29 in Japan have the heaviest burden of MDD. This may be due to the significant changes in the worldview, values, and consumption habits of the younger generation in Japan after experiencing a long 30-year economic downturn, young people in Japan have shown a downward trend in social skills, work enthusiasm, learning desire, and consumption desire, while the low desire lifestyle that emphasizes self-centeredness and laziness is increasingly being praised by Japanese young people (32, 33). The unified recruitment system for fresh graduates in Japan requires young people to quickly transition from being students to working professionals, which leads to many young people lacking autonomy in career choices and being forced to quickly enter high-intensity and highly competitive work environments. The overtime culture and strict hierarchical system in the Japanese workplace further exacerbate the psychological burden on young people aged 20–29 (34). At the same time, the proportion of informal employment in Japan (such as dispatched workers and temporary workers) is increasing, which causes Japanese young people aged 20–29 who have just entered society to face problems such as low income, little welfare, and limited career development (35). These reasons have led to the exacerbation of psychological problems in the Japanese youth population, which in turn has led to an increase in the burden of MDD among young people in Japan. The analysis of the trend of the APC age effect coefficient shows that from 1990 to 2021, except for the age group over 95 years old, the burden of MDD in all age groups in Japan showed an upward trend (local drift>0). The burden of MDD disease in the 0–15 and 60–85 age groups in Japan showed the most significant upward trend. This may be related to the enormous academic pressure on children and adolescents and population aging that is prevalent in the CJK (36). According to the trend of APC period effect coefficient changes, it can be seen that the burden of MDD in Japan from 1990 to 2005 was in a rapid upward trend, which may be related to the sustained economic downturn in Japan in the 1990s (37). In 2005, the burden of MDD in Japan showed a slow downward trend, which may be related to the fact that Japanese society has gradually adapted to the “new normal” of economic stagnation and the slowdown of economic decline since the 21st century.

The ASIR, ASPR, and ASDR of MDD in South Korea ranked second among CJK, and the AAPCs of ASIR, ASPR, and ASDR of MDD in South Korea were greater than 0 in 1990–2021. The BAPCs projections showed that the ASIR, ASPR, and ASDR of MDD in South Korea would be in an upward trend in the period of 2021–2035. Similar to Japan, the South Korean economy experienced a rapid decline after high-speed growth, which may be one of the reasons for the increased burden of MDD disease. From the 1970s to the mid-1990s, South Korea experienced a long period of rapid economic growth, and achieved the transformation from an industrial economy to a knowledge economy, which led to the world-renowned “Miracle on the Han River” (38). However, in the late 1990s, Asian countries experienced a widespread and intense financial crisis, and South Korea was one of the countries most affected by the financial crisis. After being hit by the Asian financial crisis, South Korea accepted financial relief from the International Monetary Fund and carried out the “New Economic Liberalism Reform” according to its recommendations. South Korea declared itself out of the “financial crisis” in just 2 years (39). However, the economic crisis turned into a social crisis, with job opportunities for young people continuously decreasing, lifelong employment systems being broken, class stratification, and income polarization becoming increasingly severe (40). These problems have led to the heaviest burden of MDD among young people aged 25–29 in South Korea in all age groups. According to the analysis of the trend of APC age effect coefficient changes, from 1990 to 2021, the burden of MDD in all age groups in South Korea showed an upward trend (local drift greater than 0 in all age groups), with the most significant increase in MDD burden among children and adolescents aged 0–20 and older adult people aged 80 and above in South Korea. The huge academic burden may be an important reason for the increased burden of MDD disease among South Korean children and adolescents. At present, the parents of South Korean teenagers are mostly the generation who achieved social status improvement through hard work during the “Han River Miracle” period. This generation attaches great importance to investing in their children’s education. Faced with the current situation of reduced social mobility and shrinking middle-class size in Korea, these parents are worried that their children’s social status will decline and constantly increase their investment in education and training. This has led to fierce competition in education in Korea, and the culture of tutoring is very popular (41). At the same time, South Korea has completed its economic take-off in just a few decades, but its social culture has failed to adapt to economic development at the same time. The traditional Confucian culture in South Korea emphasizes family responsibility and social obedience, but the younger generation pursues individualism more under the influence of economic development and Globalization. Cultural conflicts have led to a huge tear in South Korea’s intergenerational values (42). Economically, the economic resources of South Korea are highly concentrated in the hands of the chaebol, and the survival of small and medium-sized enterprises is difficult, which leads to the contraction of the youth employment market (43). The Gini coefficient of South Korea in 2017 was 0.36, which indicates inequality in the income of the society. South Korea’s Gini coefficient shows income inequality close to that of the United States, the highest among the OECD countries. This accelerating inequality is recognized as one of South Korea’s most serious social problems. The huge cultural differences, economic pressure, and income gap have led to a huge psychological burden on young people aged 20–29 in South Korea (44). In 2023, South Korea’s national total fertility rate was 0.72, the lowest in the world (45), South Korea’s older adult aged 65 years and older accounted for 19.2% of the country’s population (46), and the proportion of South Korea’s older adult population living alone has been increasing since 2000 (from 16% in 2000 to 22.1% in 2024) (47). Under the dual factors of declining birth rates and aging, the burden of MDD among older adult people in South Korea is also constantly increasing.

In terms of gender, the incidence, prevalence, and DALYs were higher in females than in males in all age groups in CJK during 1990–2021. Physiologically, females experience periods of intense hormonal changes such as puberty, postpartum, premenstrual, and menopause, and changes in estrogen and progesterone are closely related to the onset of depression (48). In terms of social background, East Asian women often have to maintain competitiveness in the workplace while taking on more family and parenting responsibilities. Moreover, in terms of culture, East Asian countries have a deep patrilineal cultural tradition, influenced by Confucian culture, and the concepts of “male superiority and female inferiority” and “male preference for sons” have persisted in East Asia for thousands of years. Although the status of women in East Asia has improved in modern times, achieving true gender equality is still a long and arduous process.

The risk factors that top the list of level 2 and level 3 risk factors for MDD in CJK were childhood sexual abuse and bullying and bullying victimization, respectively. Although most MDD occurs in adulthood, most of the associated risk factors begin in childhood. Most children who are bullied do not want to share their experiences with their parents or teachers for fear of retaliation or shame and therefore choose to suffer in silence, which results in bullying often being ignored by parents and teachers (49, 50). According to the “Report on the Development of Emergency Education and Campus Safety in China,” in 2016, 2017, and 2018, bullying incidents in Chinese schools accounted for 11, 24.75, and 35% of campus safety incidents, respectively (51). This shows that bullying among children is not uncommon, and the phenomenon of bullying among children is becoming increasingly serious. Therefore, timely investigation of potential conflicts and disputes between children and adolescents, and correction of their misconduct in accordance with laws and regulations, are of great significance for reducing the burden of MDD.

The burden of MDD in CJK has both similarities and differences, which requires the governments of the three countries to formulate policies that are tailored to local conditions. For China, strictly implementing the Labor Law and safeguarding the legitimate rights and interests of employees is particularly important for reducing the burden of depression among middle-aged people. Although China has achieved remarkable success in poverty alleviation in recent years, the success of poverty alleviation has narrowed the gap in infrastructure, and medical care between economically developed and underdeveloped regions. However, there are still significant differences in medical security levels between urban and rural areas and different occupations. Therefore, improving the medical security level in vast rural areas of China and for occupations such as farmers and migrant workers is also an important measure to reduce the burden of MDD in China. For Japan, it is necessary to reduce the promotion of the “overtime culture,” minimize overwork, limit overtime hours, and ensure that employees have sufficient rest time. At the same time, Japanese schools should set up dedicated psychological counseling teachers, regularly conduct mental health courses, establish student psychological records, provide early intervention for students’ depression, and actively guide young Japanese people to integrate into society. For South Korea, For South Korea, it is very important to actively reform the education evaluation system and reduce excessive reliance on exam results. In terms of economy, South Korea should strengthen the enforcement of anti-monopoly laws, crack down on and sanction the monopolistic behavior of chaebols, guide them to carry out business restructuring, and encourage innovation and development of small and medium-sized enterprises through policy support and resource allocation, reduce the excessive monopoly of chaebols on the economy, and provide more employment and promotion opportunities for young people.

This study also has some limitations. For example, due to the impact of COVID-19, there may be some bias in the data. At the same time, this study relied on a single GBD 2021 database, and the results may be biased.

## 5 Conclusion

Overall, CJK have similar cultural backgrounds, and geographical locations, and are all affected by population aging and economic fluctuations. There are many similarities in the burden of MDD, but there are also some differences in the burden of MDD disease among CJK due to economic development trends, ideological concepts, population mobility, national systems, and other factors. At present, the burden of MDD in CJK is lower than the global average, and compared to Japan and South Korea, China has a lighter burden of MDD. However, in the prevention and control of MDD, CJK still face enormous challenges.

## Data Availability

The raw data supporting the conclusions of this article will be made available by the authors, without undue reservation.

## Footnotes

^1^ https://analysistools.cancer.gov/apc/
